# Supplementary material for: Accelerating ice flow at the onset of the Northeast Greenland Ice Stream
Source: Nat Commun. 2022 Sep 23;13:5589. doi: 10.1038/s41467-022-32999-2 (PMC9508143; doi:10.1038/s41467-022-32999-2)
Supplement: Supplementary file 1 — Supplementary Information [file 41467_2022_32999_MOESM1_ESM.docx]

Online methods for

Accelerating ice flow at the onset of the Northeast Greenland ice stream

Aslak Grinsted, Christine S. Hvidberg, David A. Lilien, Nicholas M. Rathmann, Nanna B. Karlsson, Tamara Gerber, Helle Astrid Kjær, Paul Vallelonga, and Dorthe Dahl-Jensen

Correspondence to: [aslak@nbi.ku.dk](mailto:aslak@nbi.ku.dk)

## Methods 1: Velocity map trends

The ITS_LIVE dataset^14^ has annual velocity maps for the period from 1985-2018, based on optical feature tracking of Landsat images. ITS_LIVE data is distributed as files with the x and y velocity components ($v_{x}$*,* $v_{y}$). There is an associated file with the estimated errors for every pixel ($ve$). The annual maps are an average based on multiple estimates throughout the year, and thus the temporal coverage can vary from pixel to pixel. For this reason, the velocity maps are also distributed with a central date for every pixel (*t*). We down sample all data files to 1 km resolution using averaging to guard against georeferencing errors, and trends that may be caused by improving sensor resolution. This flat averaging of errors implicitly assumes fully covariant errors in a 1 km neighborhood. Further, we disregard any grid point with less than 10 years of data.

We derive accelerations from ITS-LIVE by fitting linear models of the x and y velocities as a function of years since 2000 (*t*) such as$v_{x}=a_{x}t+v_{x0}$, using least squares regression. The along-flow acceleration is obtained by projecting the acceleration $(a_{x},a_{y})$onto flow direction $(v_{x0},v_{y0})$. The results shown in the main manuscript are calculated using unweighted regression.

For every location on the map, we fit straight-line models of the form

$v_{x}=a_{x}\left( t-2000 \right)+v_{x0}$ ,

to each of the two velocity components. From this we calculate the along-flow acceleration (*a*) as

$a=\frac{{a_{x}v}_{x0}+a_{y}v_{y0}}{\sqrt{v_{x0}^{2}+v_{y0}^{2}}}$ .

We use simple linear least squares regression to estimate the coefficients ($a_{x}$, $a_{y}$*,* $v_{x0}$*,* and $v_{y0}$). We can use weighted regression to assign less weight to the data with the largest reported errors. However, this comes at a cost of introducing a temporal bias as the most modern data will inevitably have the smallest errors. For that reason, we perform the analysis using both weighted and unweighted regression. For the weighted regression for the observations from the *i*-th year, we use the following weights

$w_{i}=\frac{1}{\sqrt{ve_{i}^{2}+\left( 5\frac{m}{yr} \right)^{2}}}$.

Here, we allow for an additional 5 m/yr standard error over the reported errors ITS_LIVE. This value is comparable to the smallest reported values of *ve* in the 1980s. The linear least squares procedure also yields standard error of the coefficients ($\sigma_{ax},\sigma_{ay}, \sigma_{vx0}, \sigma_{vy0}$). Uncertainties in the intercepts are generally small relative to the intercept and are disregarded. Thus, we estimate the uncertainty in the along flow acceleration as

$\sigma_{a}=\sqrt{\frac{{\sigma_{ax}^{2}v}_{x0}^{2}+\sigma_{ay}^{2}v_{y0}^{2}}{v_{x0}^{2}+v_{y0}^{2}}}$.

In the paper we report the unweighted regression as this minimizes temporal bias. This allows for a more even temporal coverage and thus a fairer comparison between regions which we feel is important when we display it in map form.

In the resulting acceleration maps (Fig S1) we can visually discern an acceleration signal in the shear margins. However, the pointwise acceleration signal is in the same order of magnitude as the estimated standard error. The spatial coherence helps us see the large-scale acceleration pattern but is this significant. We can use averaging to reduce noise to get an improved signal to noise ratio. We therefore align the unweighted acceleration data according to the distance to the southeastern shear margin so that we can calculate an average cross profile of the acceleration pattern. The standard error of the average is calculated from the standard deviation and the effective degrees of freedom. We use the AR1 coefficient to estimate the decorrelation length scale and thus the effective degrees of freedom. The average cross profile is shown in Fig. S3.

## Methods 2: Bayesian stake analysis

We derive velocities and accelerations from repeat surveys of a stake network located around the EastGRIP camp. Second order polynomials were fitted to the GPS stake positions to derive accelerations using a Bayesian (hierarchical modelling approach. The stakes are Lagrangian and follow along with the flow, and we have therefore accounted for advective acceleration estimated from remote sensing velocities to isolate the Eulerian acceleration.

In this study, we analyze a network of 21 stakes near the EastGRIP deep drilling site (75°38’ N, 36°00’ W, 2700 masl) which we have re-surveyed every year from 2015-2019. The GPS processing is detailed in Hvidberg et al. (*15*). We estimate the velocity and acceleration of the stakes, and quantify uncertainties using PYMC3 (*21*). Our model is essentially fitting a second order polynomial to the x and y positions while accounting for a small offset that may have been introduced when stakes were extended. In the following we describe the model for the x-components only; an identical model is used for the y-components.

The GPS processing provides us with a position ($x_{gps,j}$), and an associated uncertainty ($\sigma_{xgps,j}$), where *j* is an index into one of the total 611 observations. Each observation is associated with a timestamp ($t_{j}$; years since 2017-1-1), and stake index $i_{j}$. The stakes get buried over time, and eventually they must be extended which may introduce a small unknown horizontal offset ($\Delta x_{offset,i}$) if the extension is not perfectly vertical. We encode whether an observation is from an extended stake into a binary value $h_{j}$. Our model for the stake position can be written

$x_{m,j}=x_{0,i}+{v_{x,i}t}_{j}+\frac{1}{2}\frac{Dv_{x,i}}{Dt}t_{j}^{2}+h_{j}\Delta x_{offset,i}$,

where $x_{0,i}$, and $v_{x,i}$ are the unknown position and velocity at time zero, and $\frac{Dv_{x,i}}{Dt}$ is the co-moving acceleration of the *i*-th stake. We expand the Lagrangian acceleration into components as

$\frac{Dv_{x,i}}{Dt}=a_{x,i}+\left. \frac{\partial v_{x}}{\partial x} \right|_{i}v_{x,i}+\left. \frac{\partial v_{x}}{\partial y} \right|_{i}v_{y,i}$,

assuming that the advective terms can be considered constant over the short time period considered here. The spatial velocity gradients are not known precisely, and so we allow for some uncertainty in the empirically derived value as follows

$\left. \frac{\partial v_{x}}{\partial y} \right|_{i} \sim N(\mu_{xy,i}, \sigma_{xy,i})$,

where $\mu_{xy,i}$ and $\sigma_{xy,i}$ are calculated as the mean and standard deviation from a small set of remotely sensed velocity products with good resolution and low root mean square error (*15*). In particular, InSAR velocity products (*15*, *22*, *24*) were selected as these are particularly well suited for estimating spatial gradients in velocity. These were supplemented with a multi-year average velocity product (*23*). All velocity products were smoothed with a 500 m gaussian filter before the spatial gradients were calculated.

We assume that the residuals between this model and the observations follow a normal distribution and write

${x_{gps,j} \sim N(x}_{m,j},\sigma_{x,j})$.

The standard deviation $\sigma_{x,j}$ must account for both processing uncertainties and uncertainties in antenna placement when resurveying a stake ($\sigma_{placement}^{2})$.

$\sigma_{x,j}^{2}=\sigma_{xgps,j}^{2}+\sigma_{placement}^{2}$.

Note that this $\sigma_{placement}^{2}$ is the same for all stakes, and independent of direction. We assume the that the extension offset is normally distributed as follows

$\Delta x_{offset,i} \sim N\left( 0,\sigma_{offset} \right)$,

where $\sigma_{offset}$ represents how accurately we can vertically extend a stake. We use a Half Cauchy prior centered on 0.1m for this parameter:

$\sigma_{offset} \sim HalfCauchy(0.1m)$.

For the velocities we use a gaussian prior that is centered near the remotely sensed velocities ($v_{xspace,i}$), while allowing for a global bias ($\mu_{vx}$) in the remotely sensed product:

$v_{x,i} \sim N(v_{xspace,i}+\mu_{vx},\sigma_{v})$.

The residual variance must be estimated from data but we assign a prior

$\sigma_{v} \sim HalfCauchy\left( 1 myr^{-1} \right)$

Similarly, for the accelerations we assume:

$a_{x,i} \sim N\left( 0,\sigma_{a} \right)$

$\sigma_{a} \sim HalfCauchy(0.05 myr^{-2})$

We assign a wide prior to the position at t=0, centered on the position derived in a prior study^15^,

$x_{0,i} \sim N(x_{crude,i},\sigma_{0})$.

With a HalfCauchy prior on the $\sigma_{0}$

$\sigma_{0} \sim HalfCauchy(10m)$.

# Methods 3: Kinematic wave theory

Kinematic wave theory applied to ice stream physics can give us decay length scales for different forcing periods^16^. Here, we assume a basally resisted ice stream with Weertman sliding. In this paper we apply it to a case where the slope is 0.002, thickness of 1000m, a velocity is 350 m/yr, and an ice stiffness parameter of 10^6^ Pa yr^1/3^. These values are chosen to represent a location ~100 km upstream from the Zachariæ grounding line, where we are confident that the observed dynamical changes (Fig.1) are a response to recent changes at the front^12^. The frontal changes at Zachariæ started around ~20 years ago, and thus we focus on the response to a decadal forcing time scale. With these assumptions we calculate a rapidly decaying amplitude characterized by a decay length of 57 km. I.e. at EastGRIP ~400 km upstream, we would expect a factor ~1000 reduction in the amplitude — from 2 m/yr^2^ to a practically undetectable 2 mm/yr^2^.

# Methods 4: Idealized flow model

We investigate the modelled surface velocity response to various changes in boundary conditions. Our model is a 2D cross section of an idealized, infinitely long ice stream resting on a sloping plane as illustrated in Fig. S4. The problem is reduced to two dimensions by specifying that the downhill gradient of the velocity is zero. This is equivalent to modelling the flow of a block of ice with periodic boundary conditions. The ice is assumed to be incompressible and has a canonical isotropic Glen rheology with a n=3 flow exponent and a rate factor of A=10^25^ s^-1^ Pa^-3^. We solve the stress balance using the FEniCS finite element modelling framework by closely following Rathmann and Lilien Rathmann and Lilien^25^.

We perform a total of four different experiments with the 2D cross profile model. One control experiment and three experiments where we perturb conditions slightly and observe how surface velocities change relative to the control. In the control experiment, the model domain is 150 km wide and 2000 m thick. Ice is frozen at the base (no-slip) outside of a 50 km wide slippery central region characterized by a linear Weertman sliding law with a friction coefficient of 10^3^ Pa yr m^-1^. The friction coefficient was chosen to get surface velocities that are in range of those observed at EastGRIP. At the lateral boundaries we impose $u_{x}=0$. According to ice-core observations^26^, shear margins tend to be characterized by a horizontal single maximum fabric that is favourable for horizontal xz-shear^26^. Additionally, shear heating in the shear margins could lead to further softening^3^. We therefore apply an enhancement factor of 5 in a narrow region around the shear margin in the following manner

$E\left( x \right)=1+4e^{-\frac{1}{2} \left( \frac{\left| x \right|-24km}{2km} \right)^{2}}$.

In the “*wider_slip*” experiment we increase the width of the slippery region from 50 km to 50.1 km. In the “*softer_margin*” we increase *E* from 5 to 5.2 in the shear margin. Finally, in the “*thicker*” experiment we increase the ice thickness from 2000 to 2010 m.

The results are shown in Fig. S5. Greater thickness leads to greater velocities everywhere, but in particular inside the ice stream. This is inconsistent with the observed pattern of acceleration (Fig S3), and we therefore argue that a recent increase in accumulation rates is unlikely to be the forcing mechanism. Widening the region of sliding does lead to an acceleration, but the peak is located just outside the location of maximal shear (Fig S5), in contrast to observations (Fig S3). Softening the shear margins, however, leads to an acceleration with a peak just inside the shear margin. We therefore argue that this is the most plausible mechanism for the acceleration pattern we observe. The softening could be due to shear heating or evolving fabric.

We note that we obtain compatible results when using a more modern anisotropic extension of Glen’s flow law which allows us to apply the enhancement specifically to xz-shear^29^. The results are also robust to different choices of the exact location and scale of shear margin enhancement E(x).


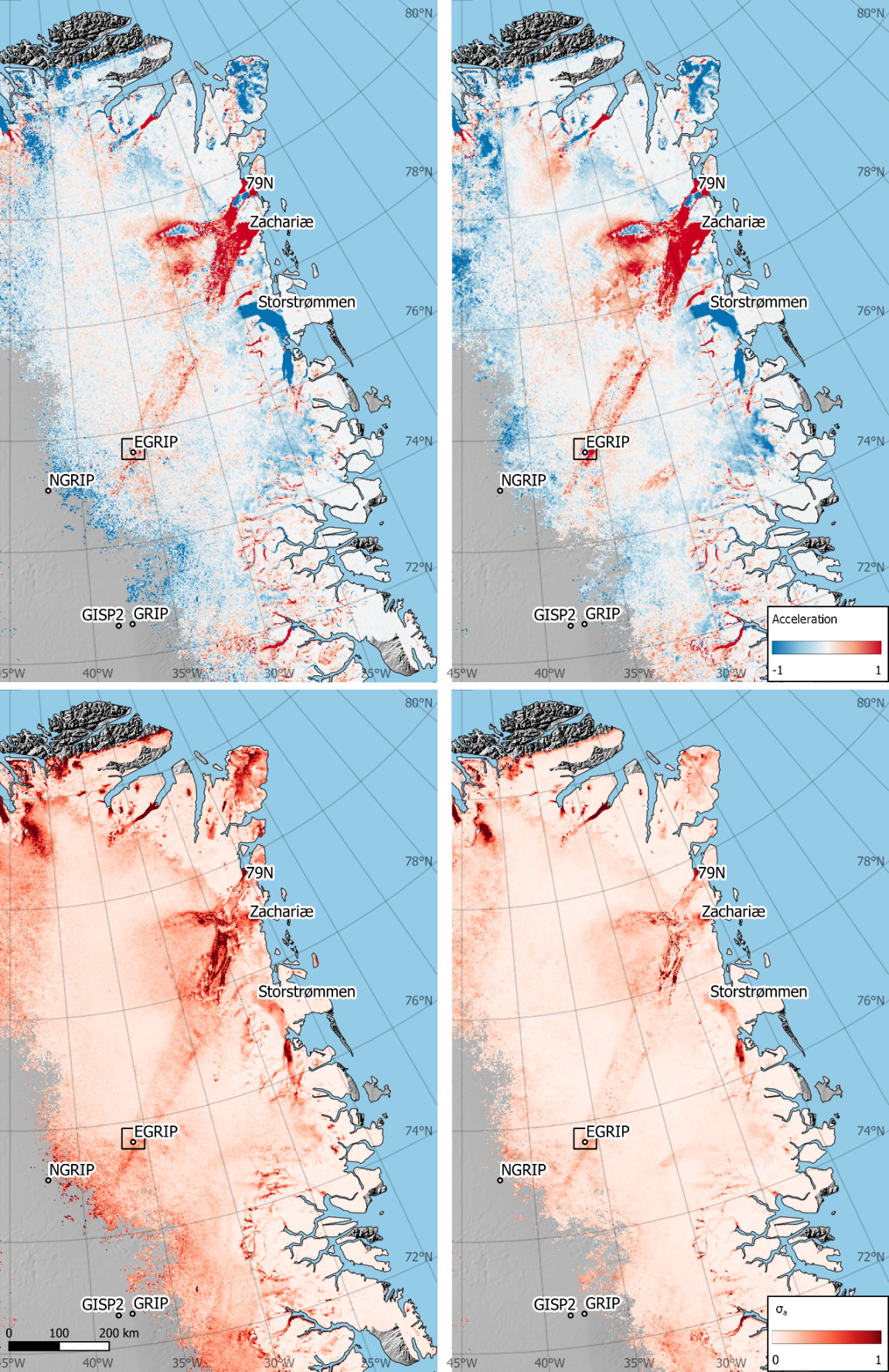


**Fig. S1.** Along flow acceleration (top) and estimated standard uncertainty (bottom) using unweighted regression (left) vs weighted regression (right). Units of all panels are m/yr^2^.


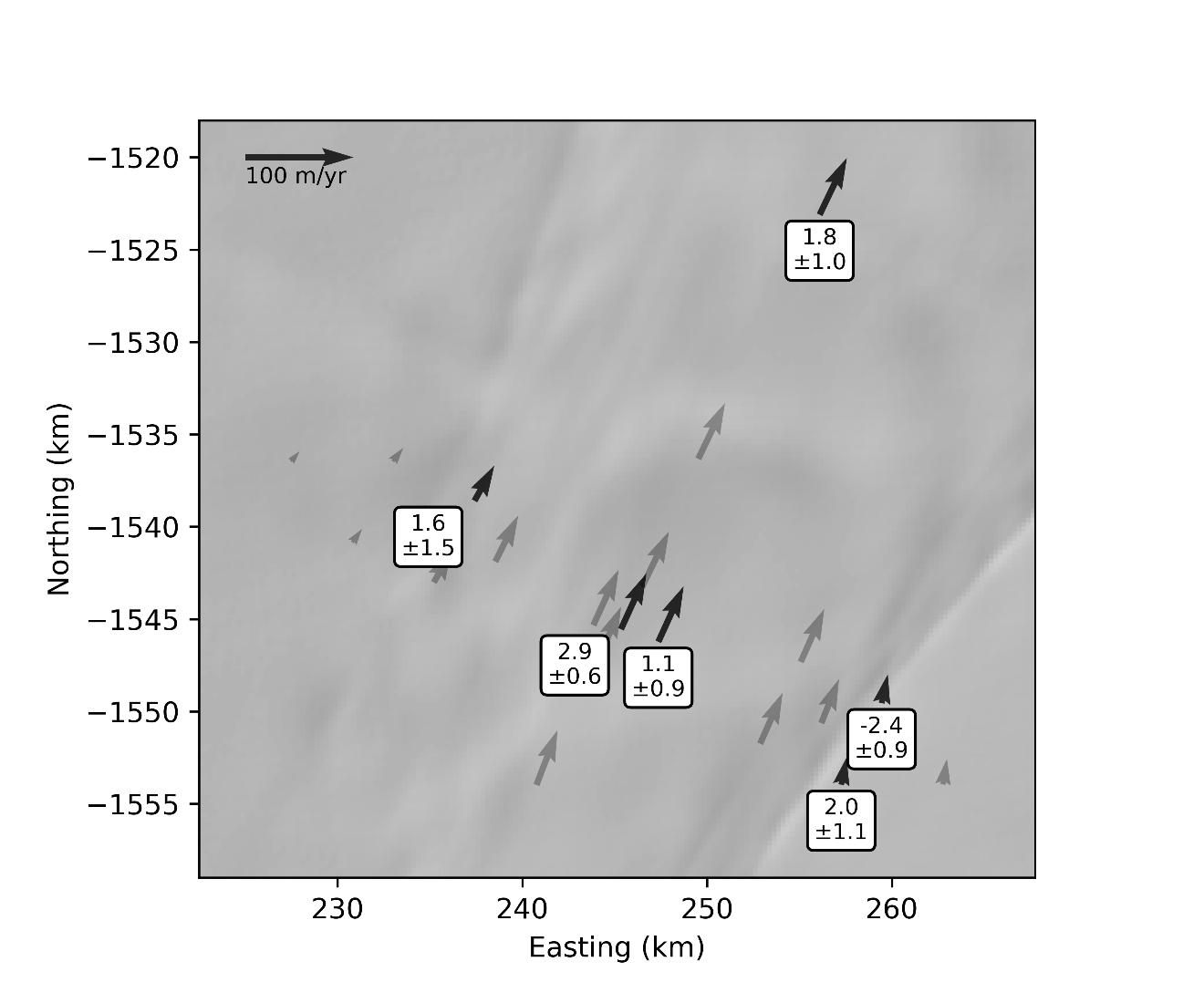


**Fig. S2.** GPS observations of stakes at EastGRIP show accelerating ice flow. Arrows indicate the velocity of each stake. Boxes at darker shaded stakes show the estimated acceleration with estimated standard deviation in cm/yr².


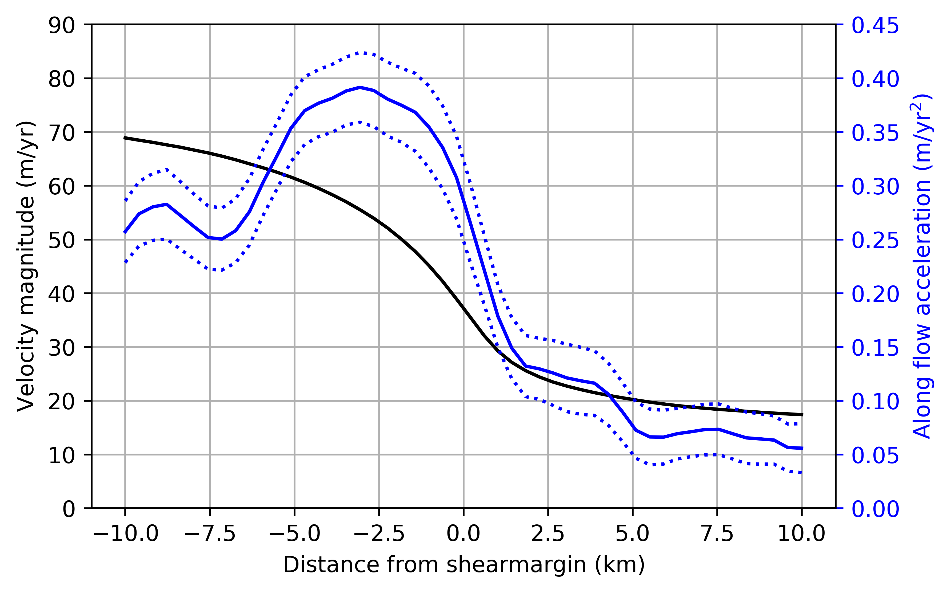


**Fig. S3.** Average cross profile of the acceleration (blue) and velocities (black) in the southeastern shear margin. The source data is the unweighted acceleration data (Fig. S1) averaged from EastGRIP to 220km downstream. Dashed line shows the standard error of the average taking an estimated decorrelation length scale of 4km into account.


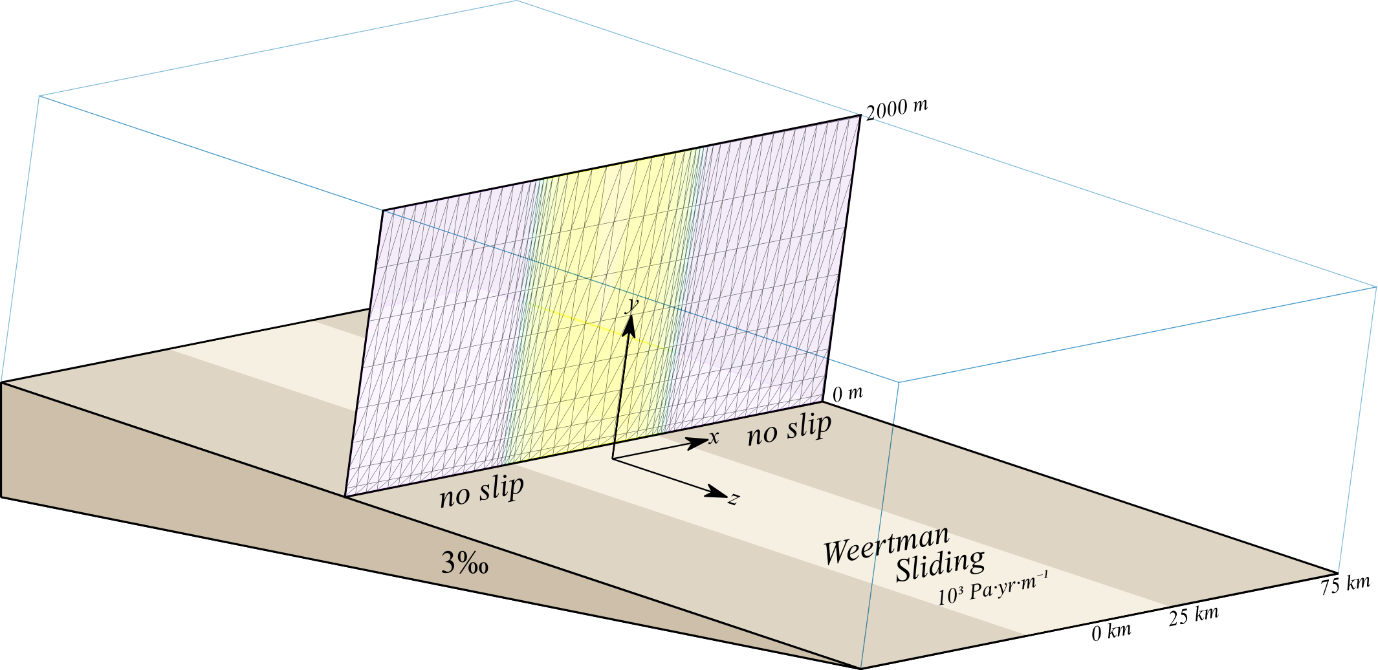


**Fig. S4. S**ketch of the idealized model of an ice stream. The model is a 2D cross section of an idealized, infinitely long ice stream resting on a sloping plane. Outside the ice stream we impose a no slip condition, and inside we use a Weertman sliding law.


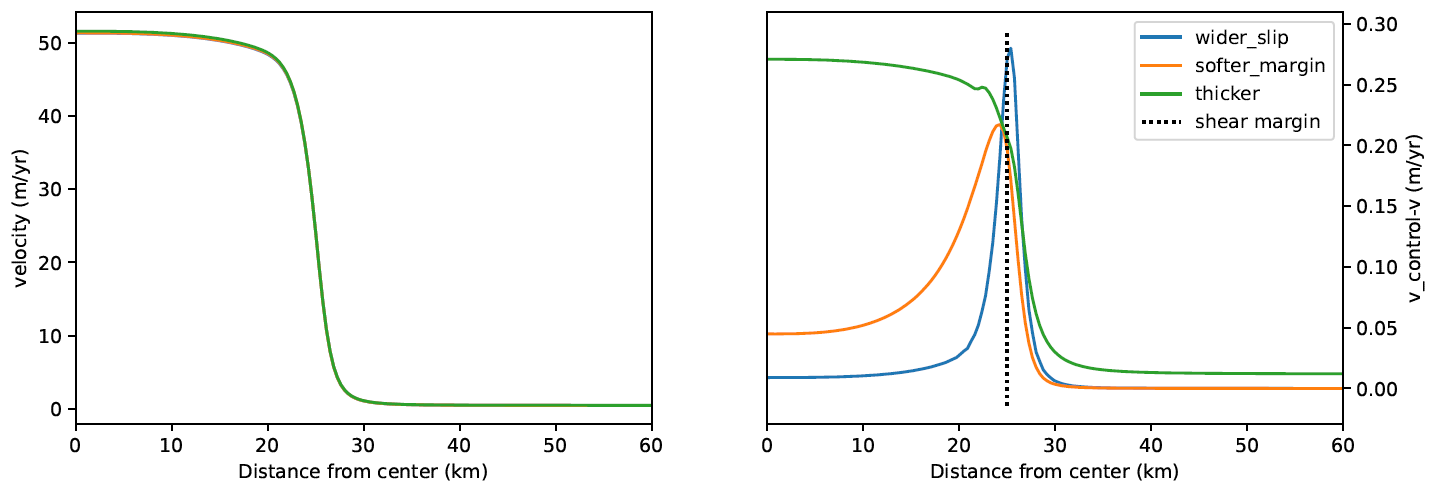


**Fig. S5.** Modelled surface velocities (left) in a 2d cross section of an idealized ice stream (see Fig. S4) for four different experiments. The difference relative to the control run is shown on the right. The ‘softer_margin’ response is most like the observed pattern of acceleration (Fig S3).

# References

1. Fox-Kemper, B., H.T. Hewitt, C. Xiao, G. Aðalgeirsdóttir, S.S. Drijfhout, T.L. Edwards, N.R. Golledge, M. Hemer, R.E.Kopp, G. Krinner, A. Mix, D. Notz, S. Nowicki, I.S. Nurhati, L. Ruiz, J.-B. Sallée, A.B.A. Slangen, and Y. Yu, 2021: Ocean, Cryosphere and Sea Level Change. In Climate Change 2021: The Physical Science Basis. Contribution of Working Group I to the Sixth Assessment Report of the Intergovernmental Panel on Climate Change [MassonDelmotte, V., P. Zhai, A. Pirani, S.L. Connors, C. Péan, S. Berger, N. Caud, Y. Chen, L. Goldfarb, M.I. Gomis, M. Huang, K. Leitzell, E. Lonnoy, J.B.R. Matthews, T.K. Maycock, T. Waterfield, O. Yelekçi, R. Yu, and B. Zhou (eds.)]. Cambridge University Press. In Press.
2. Larsen, N.K., Levy, L.B., Carlson, A.E. et al. Instability of the Northeast Greenland Ice Stream over the last 45,000 years. Nat Commun 9, 1872 (2018). <https://doi.org/10.1038/s41467-018-04312-7>
3. Holschuh, N., Lilien, D. A., & Christianson, K. (2019). Thermal weakening, convergent flow, and vertical heat transport in the Northeast Greenland Ice Stream shear margins. Geophysical Research Letters, 46(14), 8184-8193.
4. Jacobson, H. P., & Raymond, C. E. (1998). Thermal effects on the location of ice stream margins. Journal of Geophysical Research, 103(B6),12,111–12,122, <https://doi.org/10.1029/98JB00574>
5. Minchew, B. M., Meyer, C. R., Robel, A. A., Gudmundsson, G. H., & Simons, M. (2018). Processes controlling the downstream evolution of ice rheology in glacier shear margins: case study on Rutford Ice Stream, West Antarctica. Journal of Glaciology, 64(246), 583–594. <https://doi.org/10.1017/jog.2018.47>
6. Smith-Johnsen, S., de Fleurian, B., Schlegel, N., Seroussi, H., and Nisancioglu, K.: Exceptionally high heat flux needed to sustain the Northeast Greenland Ice Stream, The Cryosphere, 14, 841–854, https://doi.org/10.5194/tc-14-841-2020, 2020.
7. Mouginot, J. et al. Fast retreat of Zachariæ Isstrøm, northeast Greenland. Science 350, 1357–1361 (2015).
8. Khan, S. A. et al. Sustained mass loss of the Northeast Greenland ice sheet triggered by regional warming. Nat. Climate Change 4, 292–299 (2014). doi:10.1038/nclimate2161
9. Rathmann, N. M., C. S. Hvidberg, A. M. Solgaard, A. Grinsted, G. H. Gudmundsson, P. L. Langen, K. P. Nielsen, and A. Kusk (2017), Highly temporally resolved response to seasonal surface melt of the Zachariae and 79N outlet glaciers in northeast Greenland, Geophys. Res. Lett., 44, doi:10.1002/2017GL074368.
10. Lu An, Eric Rignot, Michael Wood, Josh K. Willis, Jérémie Mouginot, Shfaqat A. Khan (2021), Ocean melting of the Zachariae Isstrøm and Nioghalvfjerdsfjorden glaciers, northeast Greenland, PNAS, 118 (2) e2015483118; DOI: 10.1073/pnas.2015483118
11. Reeh, N., Bøggild, C. E., & Oerter, H. (1994). Surge of Storstrømmen, a large outlet glacier from the inland ice of north‐east Greenland. Grønlands Geologiske Undersøgelses, Rapp, 162, 201–209.
12. Mouginot, J., Bjørk, A. A., Millan, R., Scheuchl, B., & Rignot, E. (2018). Insights on the surge behavior of Storstrømmen and L. Bistrup Brae, Northeast Greenland, over the last century. Geophysical Research Letters, 45(20), 11-197.
13. Choi, Y., Morlighem, M., Rignot, E., Mouginot, J. & Wood, M. Modeling the response of Nioghalvfjerdsfjorden and Zachariae Isstrøm Glaciers, Greenland, to ocean forcing over the next century. Geophys. Res. Lett. 44, 071–011,079 (2017).
14. Gardner, A. S., M. A. Fahnestock, and T. A. Scambos, 2019 [Aug12 2020]: ITS_LIVE Regional Glacier and Ice Sheet Surface Velocities. Data archived at National Snow and Ice Data Center; doi:10.5067/6II6VW8LLWJ7
15. Hvidberg, C. S., Grinsted, A., Dahl-Jensen, D., Khan, S. A., Kusk, A., Andersen, J. K., Neckel, N., Solgaard, A., Karlsson, N. B., Kjær, H. A., and Vallelonga, P.: Surface velocity of the Northeast Greenland Ice Stream (NEGIS): Assessment of interior velocities derived from satellite data by GPS, The Cryosphere Discuss., https://doi.org/10.5194/tc-2020-103, 2020.
16. Williams, C. R., Hindmarsh, R. C., & Arthern, R. J. (2012). Frequency response of ice streams. Proceedings of the Royal Society A: Mathematical, Physical and Engineering Sciences, 468(2147), 3285-3310. <https://doi.org/10.1098/rspa.2012.0180>
17. Karlsson, N. B. and Dahl-Jensen, D.: Response of the large-scale subglacial drainage system of Northeast Greenland to surface elevation changes, The Cryosphere, 9, 1465–1479, https://doi.org/10.5194/tc-9-1465-2015, 2015
18. Box, J. E., Cressie, N., Browich, D. H., Jung, J-H., van den Broeke, M., van Angelen, J. H., Forster, R. R., Miege, C., Mosly-Thompson, E., Vinther, B. M., & McConnell, J. R. (2013). Greenland Ice Sheet Mass Balance Reconstruction. Part I: Net Snow Accumulation (1600-2009). Journal of Climate, 26(11), 3919-3934. <https://doi.org/10.1175/JCLI-D-12-00373.1>
19. Karlsson, N., Razik, S., Hörhold, M., Winter, A., Steinhage, D., Binder, T., & Eisen, O. (2020). Surface accumulation in Northern Central Greenland during the last 300 years. Annals of Glaciology, 61(81), 214-224. doi:10.1017/aog.2020.30
20. Franke, S., Jansen, D., Binder, T., Dörr, N., Helm, V., Paden, J., . . . Eisen, O. (2020). Bed topography and subglacial landforms in the onset region of the Northeast Greenland Ice Stream. Annals of Glaciology, 61(81), 143-153. doi:10.1017/aog.2020.12
21. Salvatier J., Wiecki T.V., Fonnesbeck C. (2016) Probabilistic programming in Python using PyMC3. PeerJ Computer Science 2:e55 DOI: 10.7717/peerj-cs.55.
22. Andersen, J. K., Kusk, A., Boncori, J. P. M., Hvidberg, C. S., and Grinsted, A.: Improved Ice Velocity Measurements with Sentinel-1 TOPS Interferometry, Remote Sens., 12, 2014, https://doi.org/10.3390/rs12122014, 2020.
23. Joughin, I., Smith, B. E., and Howat, I. M.: A complete map of Greenland ice velocity derived from satellite data collected over 20 years, J. Glaciol., 64, 1–11, https://doi.org/10.1017/jog.2017.73, 2018
24. Joughin, I., B. Smith, I. Howat, and T. Scambos. 2015, updated 2018. MEaSUREs Greenland Ice Sheet Velocity Map from InSAR Data, Version 2. [Indicate subset used]. Boulder, Colorado USA. NASA National Snow and Ice Data Center Distributed Active Archive Center. doi:10.5067/OC7B04ZM9G6Q. [Data accessed 16 Aug 2019].
25. Rathmann, N. M., & Lilien, D. A. (2022). Inferred basal friction and mass flux affected by crystal-orientation fabrics. Journal of Glaciology, 68(268), 236-252.
26. Thomas, R. E., Negrini, M., Prior, D. J., Mulvaney, R., Still, H., Bowman, M. H., ... & Lutz, F. (2021). Microstructure and crystallographic preferred orientations of an azimuthally oriented ice core from a lateral shear margin: Priestley Glacier, Antarctica. Frontiers in Earth Science, 9.
27. Joughin, I., Tulaczyk, S., Bindschadler, R., & Price, S. F. (2002). Changes in West Antarctic ice stream velocities: observation and analysis. Journal of Geophysical Research: Solid Earth, 107(B11), EPM-3.
28. Bougamont, M., Christoffersen, P., Price, S. F., Fricker, H. A., Tulaczyk, S., & Carter, S. P. (2015). Reactivation of Kamb Ice Stream tributaries triggers century‐scale reorganization of Siple Coast ice flow in West Antarctica. Geophysical Research Letters, 42(20), 8471-8480.
29. Rathmann, N. M. & D. A. Lilian (2022). On the nonlinear viscosity of the orthotropic bulk rheology. Journal of Glaciology 1–6. https://doi.org/10.1017/jog.2022.33
